# The Terebridae and teretoxins: Combining phylogeny and anatomy for concerted discovery of bioactive compounds

**DOI:** 10.1186/1472-6769-10-7

**Published:** 2010-09-17

**Authors:** Nicolas Puillandre, Mandë Holford

**Affiliations:** 1 UMR 7138, Museum National d'Histoire Naturelle, Departement Systematique et Evolution, CP26, 57 rue Cuvier, 75231 Paris Cedex 05, France; 2 The City University of New York-York College and The Graduate Center, The American Museum of Natural History NYC, USA

## Abstract

The Conoidea superfamily, comprised of cone snails, terebrids, and turrids, is an exceptionally promising group for the discovery of natural peptide toxins. The potential of conoidean toxins has been realized with the distribution of the first *Conus *(cone snail) drug, Prialt (ziconotide), an analgesic used to alleviate chronic pain in HIV and cancer patients. Cone snail toxins (conotoxins) are highly variable, a consequence of a high mutation rate associated to duplication events and positive selection. As *Conus *and terebrids diverged in the early Paleocene, the toxins from terebrids (teretoxins) may demonstrate highly divergent and unique functionalities. Recent analyses of the Terebridae, a largely distributed family with more than 300 described species, indicate they have evolutionary and pharmacological potential. Based on a three gene (COI, 12S and 16S) molecular phylogeny, including ~50 species from the West-Pacific, five main terebrid lineages were discriminated: two of these lineages independently lost their venom apparatus, and one venomous lineage was previously unknown. Knowing the phylogenetic relationships within the Terebridae aids in effectively targeting divergent lineages with novel peptide toxins. Preliminary results indicate that teretoxins are similar in structure and composition to conotoxins, suggesting teretoxins are an attractive line of research to discover and develop new therapeutics that target ion channels and receptors. Using conotoxins as a guideline, and innovative natural products discovery strategies, such as the Concerted Discovery Strategy, the potential of the Terebridae and their toxins are explored as a pioneering pharmacological resource.

## Introduction

**Figure 1:**
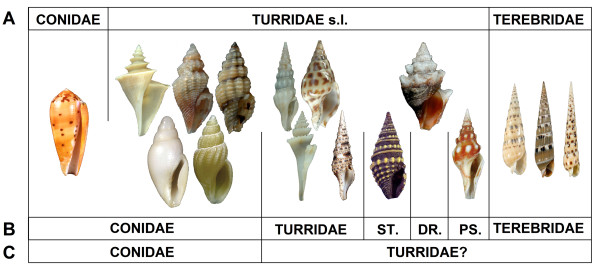
**Evolution of Conoidea classification**. Cone snail, turrid, and terebrid shells that make up the Conoidean superfamily are depicted. A. Conoidea classification based on shell and radula characters (e.g. [8,57]). B. Conoidea classification based mainly on anatomical characters ([1]). ST = *Strictispiridae*; DR = *Drilliidae*; PS = *Pseudomelatomidae*. C. Conoidea classification with molecular characters. Two main clades are defined, but a formal classification has not yet been proposed.

The conoideans (cone snails, terebrids, and turrids) are a hyperdiverse group of marine gastropods that prey on fish, worms, and other mollusks (Figure 1). Several conoidean lineages are characterized by specialized organs referred to as a venom apparatus that is used to subdue prey [1]. Analysis over the last three decades of venom toxins produced by various species in the genus *Conus *(cone snails), the most famous representative of this group, reveal a complex system of molecular compounds (see *e.g*. [2,3]). Each *Conus *species is able to produce 100-200 peptide toxins [4,5], making this genus, and by extension the whole Conoidea superfamily, one of the most promising groups for the discovery of natural peptide toxins together with snakes, spiders and scorpions.

Within the conoideans, the auger snails, or the Terebridae, include approximately 300 to 350 described species [6,7]. The Terebridae, characterized by an elongated shell, are mostly sand-dwellers that live in shallow-waters near the tropics. Contrary to cone snails, terebrids have not attracted significant scientific attention, and comparatively little is known about their ecology and toxinology. Most of the main lineages of conoideans, including terebrids and conids, diverged at least in the early Paleocene [8]. Such an early separation would indicate toxins from terebrids could be highly divergent and unique, compare to toxins found in the genus *Conus*.

Presented here is an overview of the emerging potential of terebrids and their peptide toxins. As terebrid toxins are closely related to cone snail toxins (conotoxins), what is known about the structural and functional diversity of conotoxins, and their application in pharmacology is first briefly reviewed. In addition, a comparison will be made of the traditional biochemical approach to peptide toxin discovery, and a novel multidisciplinary biodiversity first approach, termed the *Concerted Discovery Strategy (CDS)*. CDS combines molecular and chemical techniques with phylogenetic analysis of species and toxin evolution to enhance the discovery of peptidic natural products. Recent results highlight the advantage of CDS to quickly define independent lineages within *Conus *[9,10] and the terebrids [11,12], thus facilitating the identification of numerous and divergent species, each producing unique peptide toxins. By analogy with "conotoxin", the term "teretoxin" is introduced to designate natural peptide toxins produced by terebrid snails.

## 1. Conotoxins and pharmacology

### 1.1. Brief history of the discovery of conotoxins

Cone snails were known as venomous predators [13] for many years before the analysis of their venom started in the 1970's, with the isolation of active compounds from the venom gland of *C. californicus *[14] and *C. geographus *[15]. In 1981, Gray et al.[16] first biochemically described the structure and function of several conotoxins extracted from *C. geographus*. Soon after, the Olivera group identified numerous toxins from other *Conus *species, such as *C. magus*, *C. striatus*, and *C. textile *[17,18]. In the following two decades, the regularity of toxin discovery has been enhanced both by the number of laboratories working on conotoxins, and by the use of new techniques that improved characterization methods such as molecular biology, mass spectrometry, and sequencing. Currently there are more than 3,000 different proteins extracted from *Conus *venom described (Conoserver: http://research1t.imb.uq.edu.au/conoserver/).

### 1. 2. Structure and function of conotoxins

**Figure 2:**
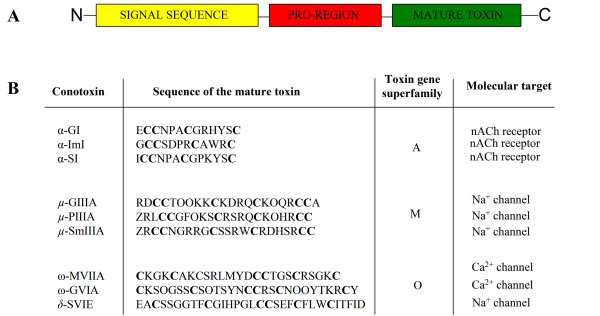
**Molecular organization of Conoidean venom toxins**. A. Schematic of the precursor sequence for conoidean toxins. Conoidean toxins possess a signal sequence at the ntermini, an intervening pro-region, followed by the mature toxin in single copy. Each gene superfamily is generally characterized by one highly conserved signal sequence, associated in most cases to one cysteine (Cys)-pattern in the mature sequence, and corresponding to several toxin families (such as α, μ, ω, δ) and molecular targets (ion channels or receptors). B. Conotoxin examples. Depicted are the mature toxin sequences, gene superfamily, and molecular targets of well characterized conotoxins.

**Figure 3:**
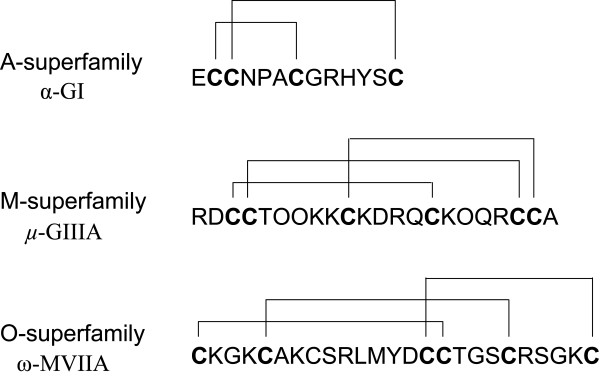
**Cysteine scaffold of conotoxins**. Representative disulfide connectivity of three conotoxins belonging to three different gene superfamilies are illustrated.

The vast majority of conotoxins are characterized by a three-domain structure consisting of: a highly conserved signal sequence, a more variable pro-region and a hypervariable mature sequence (Figure 2A). The signal sequence can be used as a diagnostic character to attribute each conotoxin to one of the ~15 superfamilies described so far (Figure 2B). The mature toxin is a disulfide-rich peptide with a highly conserved cysteine pattern in each superfamily [5] (Figure 3). At least 25 different functions have been described for a small fraction of the known conotoxins that have been characterized ([5]; conoserver). By the end of the 1990's, given the diversity of their molecular targets such as, sodium (Na^+^), potassium (K^+^) or calcium (Ca^2+^) channels, noradrenaline transporter, and nicotinic acetylcholine (nACh) receptors, it became apparent conotoxins possessed potentially numerous therapeutic applications.

### 1.3 The emergence of conotoxins for drug development

The first conotoxin to be approved for use as a drug is ziconotide (Prialt), which is used to treat chronic pain in HIV and Cancer patients [19]. Ziconotide was discovered and developed from the ω -MVIIA peptide expressed by *Conus magus *(Figure 3). As other ω conopeptides of the O superfamily, MVIIA targets Ca^2+ ^channels and has high specificity for the N-type calcium channel Ca_V_2.2. The emergence of ziconotide has led to the increased investigation of cone snail peptides in drug development. This is in large part due to the large number of conotoxins discovered and their specificity for particular ion channels and receptors. A recent review [5], listed several conotoxin-derived peptides that reached clinical development at various stages. These include: Contulakin-G (neurotensin receptor), χ -MrIA (norepinephrine transporter), α-Vc1.1 (nicotinic receptors), Conantokin-G (NMDA receptors), κ-PVIIA (K^+ ^channels), and μO-MrVIB (Na^+ ^channels). Most of the contoxins listed are potential therapeutics for pain, but several are being evaluated for epilepsy or myocardial infraction. Twede et al. [20] also cited several other conotoxins with neuroprotective/cardioprotective properties: namely, conantokins, ω, μ and κ-conotoxins that respectively target NMDA receptors, Ca2^+^, Na^+ ^and K^+ ^channels. It should be noted that although ziconotide is a breakthrough, delivery of the drug by intrathecal injection is problematic and limits its utility.

## 2. *Conus*: the tree that hides the forest

### 2.1. Conoidean phylogeny

*Conus *and the Terebridae both belong to the superfamily Conoidea. This group has always been considered a taxonomic nightmare, primarily because of its substantial diversity, 4,000 described species, with an estimate of more than 10,000 living species [21], and secondarily because of the difficulty to propose a stable system of classification [22]. Very few classifications have been proposed, however most of them are not congruent, and are largely contradictory. Conoidean classification has evolved in accordance with the character type used to delimit groups. Initially, only shell and radula characters were used. As they are beautifully ornamented and easily distinguished, cone snails are the most famous conoideans, a star among shell collectors, taxonomists, and biochemists alike. Consequently, cone snails were classified in a separate family, the Conidae. Similarly, the Terebridae, with their thin, elongated shells, are relatively easy to recognize, and were also classified as an independent family. All the others conoideans were placed in the Turridae s.l. (Figure 1A). More recently, the analysis of anatomical characters revealed that cone snails are not so different from other conoideans, and some turrids (Clathurellinae, Raphitominae, Mangeliinae, Oenopotinae, Conorbinae) were placed in the Conidae together with *Conus *([1]; Figure 1B). The use of molecular characters to analyze conoidean classification gave yet a different structure to the superfamily. Molecular characters confirmed that Turridae s.l. was a largely paraphyletic group, including *Conus*, but also Terebridae [22]. It is clear that more interdisciplinary research that combines molecular, anatomical and morphological characters is needed to establish a valid classification of the Conoidea.

### 2.2. The revolution of molecular phylogeny as it pertains to Conoideans

The advent of the polymerase chain reaction (PCR) and sequencing has revolutionized taxonomic classification. Together with anatomical and morphological characters, molecular approaches help to define distinct biodiverse groups. Molecular approaches identified at least 15 independent lineages within the conoideans, most of them corresponding to previously recognized taxa [1,8,22,23]. Some taxa were traditionally recognized as families, i.e. *Conus *as Conidae, while others were considered as subfamilies, i.e. Mangeliinae, and Crassispirinae. Molecular results suggest that the genus *Conus *does not have a central position in the superfamily, but rather it corresponds to one lineage among others. Even if cone snails remain the most collected and studied group within Conoideans, terebrids and turrids are a compelling research source as they may have evolved unique and diverse venom toxins. Preliminary analyzes of turritoxins [24,25] and teretoxins [26,27] are promising (Table 1).

Consider for a moment, if 3,000 conotoxins are already described, how many peptide toxins can be expected for the whole conoideans? How can such diversity be embraced, and how can peptide toxin discovery be optimized? In the next two sections, an estimation of peptide toxin diversity is proposed to answer the first question, and a new strategy, termed "Concerted Discovery Strategy (CDS)," is described to handle the second.

### 2.3. A sizeable natural library of peptide toxins

**Figure 4:**
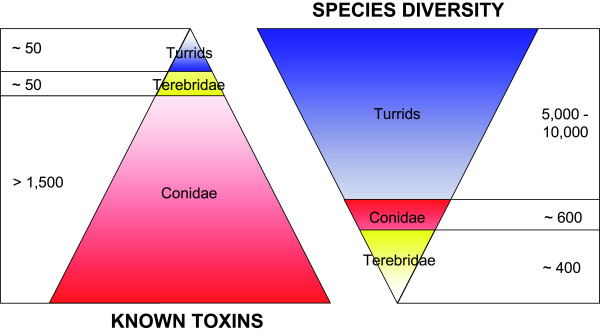
**Comparison of the known number of toxins (left) and species (right) in the main groups of the Conoidea**. The number of toxins in Conidae ("conotoxins") corresponds to the published toxins in GenBank. No turritoxins or teretoxins are published in GenBank, but several were recently described ([24-29]).

It has been shown that each *Conus *species can express between 100 and 200 different peptide toxins, most being exclusive, i.e. not found in any other species. With more than 600 described species, and others remaining to be discovered, it can estimated that 60,000 to 120,000 different peptide toxins could be produced by cone snails. These numbers are probably underestimated, as unpublished results (F. Ducancel et al., A. Lluisma and P. Bandyopadhyay) indicate that a single species may include 200 different toxins, only for the A-superfamily. Preliminary results obtained for terebrids and turrids seem to indicate that similar levels of toxin diversity occur in these two groups as well [24-29] (Figure 4). Based on these conclusions, it is possible to estimate that the whole Conoidean superfamily could contain between 400,000 and 2,000,000 different toxins. Spiders are the only other venomous group thought to include similar levels of toxin diversity [30]. Conoideans are thus producing a sizeable natural library of peptide toxins that have potential applications for biomedical applications and drug development.

### 2.4. A concerted discovery strategy for finding new peptide toxins

**Figure 5:**
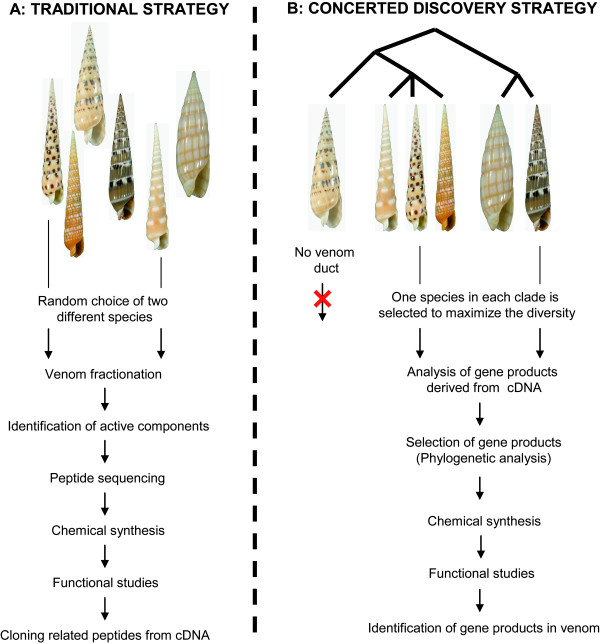
**Toxin discovery process**. A. Traditional strategy. Analyzed species are chosen randomly, and may correspond to a single lineage. Prospective toxin analysis starts first with characterization of venom components by HPLC (venom fractionation). B. Concerted Discovery Strategy (CDS). Taxonomic tools are used first to identify independent lineages to maximize the species, and thus teretoxin diversity, and then to analyze the numerous cDNA compounds isolated from each analyzed specimen. Application of CDS increases discovery of divergent teretoxins.

As applied since the beginning of the 1980's, the traditional process of toxin discovery is to fractionate the crude venom from a target species, then characterize the fractions using Edman sequencing or electrospray ionization mass spectrometry (ESI-MS/MS) (Figure 5A). Most of the species that have been studied with this method correspond to species that are easy to collect, large enough to allow an easy extraction of the venom in a sufficient quantity, and known to be highly venomous, especially for vertebrate preys. These features were thought to indicate toxins viable for therapeutic applications in humans. While overwhelmingly used by most researchers, the traditional strategy has several drawbacks, such as it is extremely laborious and requires large amounts of material to be successful. Several findings from the work of the Olivera group [31,32] in the 1990's elucidated the molecular structure of conotoxins (Figure 2). Namely, the conserved signaling region of the peptide toxin gene superfamilies has enabled the use of PCR and other molecular techniques to minimize the identification of toxins using the traditional strategy. However, in cases where a significant amount of sample material is not available, use of the traditional strategy is a challenge.

The Concerted Discovery Strategy (CDS), previously referred to as the exogenomic strategy as proposed by Olivera [5], differs from the traditional toxin discovery strategy in the way species and venom compounds are selected (Figure 5B). With CDS, species are not chosen based on technical criteria, such as size and ease of collection, but using an evolutionary-based approach. The central idea is to identify species that belong to highly divergent lineages, thus potentially able to express highly divergent toxins. This method enhances the probability of characterizing different toxins. A larger pool of different toxins increases the likelihood of identifying those with different molecular targets, indicating different therapeutic applications. Compared to the traditional strategy, where most of the studied species belonged to a limited number of clades within *Conus*, suggesting a biased estimation of the peptide toxin diversity within the genus, CDS is much more aligned with identifying a diverse set of peptide toxins with diverse functional applications. Furthermore, a biodiversity first concerted method will allow the identification of the lineages that have lost the venom apparatus within the Conoideans, a not-so-rare feature in this group (see Sect. 4.2.2, but also [1,33,34]; Fedosov and Puillandre unpublished data). *A priori *knowledge about the presence or absence of a venom apparatus eliminates the expense of time and resources pursuing specimens without venom ducts, hence not expressing peptide toxins to hunt prey.

Another feature of CDS makes use of the advances of molecular biology in the form of manufacturing cDNA or EST libraries. Using cDNA libraries of venom duct tissue, expressed gene products are analyzed, and potential peptide toxin are identified. At this stage, a phylogenetic approach can be used to analyze the toxin diversity within a single species. Recent studies have shown that divergent clades within a toxin-based phylogeny may produce toxins with different functions (*e.g*. [2,9,10,35]). Instead of functionally analyzing randomly-chosen toxins, as in the traditional method, CDS highlights promising toxins to be screened first. Using CDS, phylogenetic methods are used to identify prospective targets, first species within conoideans and then toxins within the selected species.

## 3. The Terebridae family

### 3.1. Traditional taxonomy of the Terebridae

The Terebridae was first identified and classified by Bruguiere (1789), who created the genus *Terebra*. Since that time the seminal works to classify the group have been presented in recent papers highlighting the anatomy and shell morphology [7,36-38]. Miller in his publications in the 1970's plucked the group from relative obscurity to highlight the fascinating degree of anatomical variability that accounts for the diverse feeding strategies within the Terebridae. Based on analysis of foregut materials Miller identified three different types of terebrid anatomy: (1) Type I has salivary glands, a shrunken buccal tube, no radula sac, venom duct, or venom bulb. (2) Type II has the venom apparatus similar to *Conus*, i.e. a radular sac, venom duct and venom bulb, in addition to salivary glands and a true proboscis. (3) Type III lacks salivary glands and the components of the venom apparatus, but has an uncharacteristic accessory feeding organ, the accessory proboscis structure. Using specimens from the genera *Duplicaria*, Taylor has revised terebrid foregut anatomy based on radula characteristics and identified an amendum to Miller's Type I that has salivary glands and a radula sac, but no venom gland [37]. Based on shell morphology, Bratcher and Cernohorsky [7], and more recently Terryn [6] have identified ~300 different species within the Terebridae. Bratcher and Cernohorsky placed the species into four genera: a large genus termed *Terebra*, consisting of the majority of species, a second genus termed *Hastula*, a third genus termed *Duplicaria*, and a fourth termed *Terenolla*. Terryn in his classification made use of ~15 genera terms, including *Myurella*, and *Cinguloterebra*. The first phylogeny of the group was done by Taylor et al. [1]. Using seven species of Terebrinae and seven of Pervicaciinae, Taylor and colleagues outlined anatomical terminology for the terebrid foregut and postulated a phylogeny that identified the Terebridae as monophyletic and separate from the Conidae.

Simone in 2000 [39] updated the terebrid phylogeny using specimens from the Western Atlantic. Simone confirmed the monophyly of the group, identified the *Hastula *genera as separate from the genus *Terebra *and found the following apomorphies: reduction of the cephalic tentacles, anterior end of the ctenidial vein prominent (without gill filaments), rhynchodcal introvert, and anus situated very posteriorly in the pallial cavity. Bouchet and Rocroi [40] in the most recent classification based on morphology of the Gastropoda confirmed the presence of two subfamilies, Terebrinae and Pervicaciinae, within the family Terebridae. The use of anatomy, and shell characteristics were sufficient to elucidate the monophyly of the Terebridae, but for definitive delimitations at genera and species level, an integrative approach using molecular biology is required.

### 3.2. Molecular Phylogeny applied to the Terebridae

**Figure 6:**
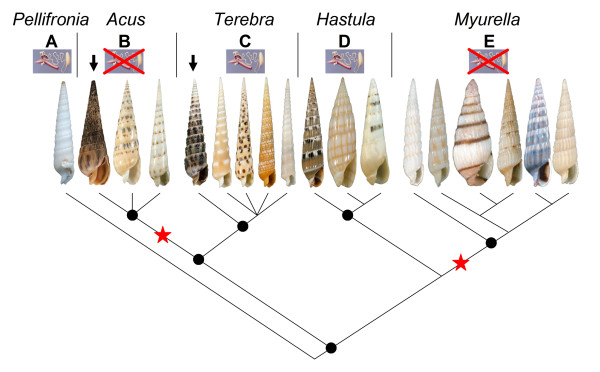
**Molecular phylogeny of the Terebridae**. Five clades are defined *Pellifronia, Acus, Terebra, Hastula, and Myurella *(Clades A to E). Two clades have lost the venom apparatus: *Acus *(clade B) and *Myurella *(clade E), corresponding to two independent losses (red stars). Two lineages are found in the Panamic Eastern Pacific, black arrows correspond to *Acus strigata *and *Terebra arygosia*, the others in the Western Pacific. The tree was constructed using Bayesian and likelihood analyzes based on COI, 12S and 16S mitochondrial genes, see [11,12] for full details on the molecular phylogeny. The molecular phylogeny of the Terebridae effectively highlights the terebrid lineages that have a venom apparatus (*Pellifronia, Terebra, Hastula*), therefore using peptide toxins to subdue prey, and those that do not have a venom apparatus (*Acus, Myurella*), thus not using toxins to hunt.

The revisionary process that resulted from the use of molecular characters for the Conoideans classification also happened for the Terebridae. Most of the genera recognized by Terryn [6] do not correspond to clades, as defined by molecular analyzes based on 16S, 12S, and COI mitochondrial genes and ~50 different species [11,12]. Molecular analysis identified five distinct clades in the Terebridae: a sister group to all other terebrids made up of *T. jungi*, since revised to the genus *Pellifronia *[41] (clade A), an *Acus *clade (clade B), a *Terebra *clade (clade C), a *Hastula *clade (clade D), and a *Myurella *clade (clade E) (Figure 6). This result indicates that most of the morphological characters used to define genus-level groups of terebrids should be used with caution, and could correspond to convergent evolution or ancestral polymorphism. The genus "*Terebra*" is a good illustration of the conflict between classical morphological characters and molecular data. Specimens morphologically attributed to this genus are found in three different clades: *Pellifronia*, *Terebra*, and *Myurella *as identified by Holford et al. [11,12].

In addition to the clarification of the phylogenetic relationships within the Terebridae, the tree outlined in Figure 6 provides a reliable framework to analyze the evolution of different terebrid characters (see Sect. 4).

### 3.3. Alpha-taxonomy of the Terebridae

As stated above, the only available molecular work on terebrids highlighted several complications at the generic level, and also revealed that the alpha-taxonomy (species delimitation and description) may need to be revised [12]. Based on shell characters only, the definition of species in the Terebridae certainly suffers from the same pitfalls cited previously for the classification: morphological convergence or ancestral polymorphism. Furthermore, the molluscan shell is known to be highly plastic, and morphological variation may only be the results of environmental variability within a single species range [42]. The results presented in Holford et al. [12] indicate that some species correspond to several lineages (*e.g. Strioterebrum plumbeum*, *Cinguloterebra fenestrata*), and that several molecularly defined lineages are not named at the species level (*e.g*. the "*Terebra*" *textilis *complex). However, the sampling used in this study does not allow a clear analysis of the species-level variability within the terebrids. More specimens are required to estimate both the intra and inter-specific variability of each putative species, in order to propose robust hypotheses of species delimitation.

Several recent expeditions carried out in the Indo-West Pacific by the Muséum National d'Histoire Naturelle (Paris) uncovered a large number of specimens, representing both already described species and several unknown taxa. Recently, four different Terebridae species were collected during an expedition in the East Pacific (Panama) [11]. Two of these four species were represented by several specimens, and the results are congruent with the morphological hypotheses: the molecular variability within species is weaker than the variability between species.

### 3.4 Terebrid ecology and behavior

Miller [43] provided the most detail known to date on the feeding ecology of the terebrids. Miller described three anatomical feeding varieties (Types I, II, and III) only one of which (Type II) possessed the venom apparatus similar to that used by *Conus*. The terebrid clades that have a venom apparatus correspond to three divergent lineages, implying the three clades with a venom apparatus (*Pellifronia, Terebra, and Hastula*) may have evolved different feeding strategy (Figure 6). Furthermore, the two other terebrid lineages that have lost the venom duct, Types I and III, while not good candidates to find new teretoxins, are good models to analyze the ecological adaptation of venom-apparatus free conoideans. Questions to be investigated include, how do species that lack the venom duct and radula, which are the main characteristics for capturing prey using venom toxins, feed? Are they still able to capture prey using toxins produced by other glands (e.g. the salivary gland), or did they develop new strategies not based on venom? If so, did the two lineages without the venom ducts develop similar or different strategies? Preliminary work in *Conus *has demonstrated that peptide toxins are produced in the salivary glands [44], suggesting it may be possible that the Type I species of terebrid, which lack a venom apparatus, but have salivary glands, could also use toxins to subdue its prey. The delivery of the toxins is not clear as most Type I species do not have a radular or a true proboscis to deliver the toxin to the prey. Type III terebrids have developed an accessory feeding organ that they use to engulf polychaetes and other worms.

There are many open questions pertaining to the ecology of the terebrids. These could be addressed in tandem with studies involving the evolutionary development of the venom apparatus. Of the three Miller types which is the ancestral one that led to the development of the others? The radula is often hailed as the component responsible for the radiation of species diversity in *Conus *[45], could it also explain diversity in Type II terebrids? Given the complications of breeding terebrids and the complexity of the venom apparatus, evolutionary development questions might be difficult to approach using embryology and current evo-devo techniques, but highlight an interesting line of research that would enhance current knowledge about the terebridae and evolutionary/ecological development in general.

### 3.5 Evolution of venom apparatus as it pertains to peptide toxins in the Terebridae

One striking result of the molecular phylogenetic analysis of the Terebridae is that the venom apparatus appears to have been lost at least twice independently during the evolution of the group (Figure 6). The corollary finding is that the venom duct was present in the common ancestor of all the terebrids, and also in the common ancestor of all the conoideans [1]. From this common ancestor, three highly divergent lineages evolved independently in the Terebridae: *Pellifronia, Terebra *and *Hastula*. As these lineages correspond to deep nodes in the tree, and given the extremely high rate of evolution of the toxins in the genus *Conus *[2,3], these terebrids may have evolved different toxins. The conotoxins discovered so far belong to ~ 15 different superfamilies, and evolved ~ 25 different functions; however, the genetic distance between *Pellifronia, Terebra *and *Hastula *within the Terebridae is at least two times greater than the distance between the different species of *Conus *from which the known conotoxins were extracted (unpublished results). The potential divergence between teretoxins extracted from species belonging to different clades suggests previously undescribed superfamilies and functions could be identified from terebrid characterizations.

### 3.6 Preliminary characterization of teretoxins

While only a few teretoxins have been described in the literature, results from preliminary characterizations indicate their potential as biochemical tools for analyzing the mechanics and function of the neuronal circuit. Several teretoxins, previously referred to as augertoxins, identified by Imperial and colleagues [26,27] from *Terebra subulata *and *Hastula hectica *have a cysteine framework similar to the O-superfamily of conotoxins (Table 1). This suggests that they may fold into the inhibitory cysteine knot motif referred to as the ICK motif [46]. The ICK motif is common among peptide toxins from various organisms including snakes and spiders, and is known to block ion channels. While the *T. subulata *teretoxins identified have a similar O-superfamily cysteine framework, the signal sequence of the precursor region is not homologous with the conotoxin O-superfamily signal sequence [26]. This suggests that although the mature toxins are similar, the genes encoding the peptides are not. Likewise, the teretoxins identified from *H. hectica *[27] have cysteine patterns similar to the O and P conotoxin superfamilies, but their signal sequences are highly divergent. These findings indicate the genetic makeup of *conus *and terebrid toxins are not the same. It thus follows that newly discovered teretoxins could have diverse functional applications compared to their conotoxin counterparts.

**Table 1:** Recently identified teretoxins.

Teretoxin	Mature Toxin Sequence	Corresponding conotoxin Superfamily	Potential Target
Agx-s11a	D**C**EQHTD**C**SAASGPVY**CC**QDSD**CC**GGVDYI**C**TNYGQ**C**VRHF	I	K+ channels

Agx-s6a	SLDEELKSND**C**PEY**C**PHGNE**CC**EHHE**C**RYDPWSRELK**C**LDSLDS	O	Na+, K+, Ca2+ Channels

Agx-s7a	ATNRHQ**C**DTNDD**C**EEDE**CC**VLVGGNVNNPGVQTRI**C**LA**C**S	O	Na+, K+, Ca2+ Channels

Hhe6.3	VLFTPPELLG**C**GNR**C**SDD**CC**KWGR**C**QPG**C**TD	O	Na+, K+, Ca2+ Channels

Hhe9.1	YEEN**C**GTEY**C**TSKIG**C**PGR**C**V**C**KEYNYNGEITRR**C**RA	P	Unknown

Hhe9.2	DEEVG**C**FPNV**C**KNDGN**C**SIETSTGMTR**C**Q**C**LEGYTGHV**C**ENPL	P	Unknown

## 4. Taxonomy as a tool for discovery of bioactive compounds

### 4.1. Congruence between anatomy and molecular phylogeny

The strong congruence between anatomy and the molecular phylogeny based on Western Pacific species is shown in Figure 6. All the species included in the *Acus *and *Myurella *clades do not have a venom apparatus, therefore, likely not using peptide toxins to hunt prey. Conversely, all the species included in the three other clades, *Pellifronia*, *Terebra *and *Hastula *all have the venom apparatus, as confirmed by the anatomical dissection of most of the species included in the dataset [12]. The correlation between anatomy and molecular phylogeny was confirmed by the inclusion of several species collected in the Eastern Pacific. The Panamic species *A. strigata*, placed in the *Acus *clade, does not have a venom apparatus, while three other Panamic species, *T. argyosia, T. ornata, and T. formosa*, which possess a venom apparatus, are placed in the *Terebra *clade [11]. These results support the premise that the presence or absence of the venom duct can be inferred by including a given species in the phylogenetic tree, without dissecting it. From a teretoxin discovery perspective, the phylogenetic tree would then be an invaluable asset, capable of readily identifying the lineages with a venom apparatus and expressing peptide toxins for predation. *A priori *identification of terebrids expressing peptide toxins enhances by several orders of magnitude the initial step of characterizing novel teretoxins. In addition, the phylogenetic tree could be used to identify divergent lineages and enhance discovery of teretoxins with different functional applications. Analysis of at least one species from each clade with a venom apparatus would be sufficient to provide a gross estimation of the toxin diversity of terebrids.

### 4.2. The importance of a complete Terebridae phylogeny for teretoxin discovery

The current molecular phylogeny of the Terebridae [11,12] is not comprehensive. Several genera and biogeographic regions are not represented in the dataset. As five lineages are present in the West Pacific, two of them having lost the venom apparatus, it can be expect that there are additional lineages in other regions that have evolved distinct toxins and possible other independent lineages that have lost the venom apparatus. A complete terebrid phylogeny would greatly enhance the discovery and characterization of novel teretoxins. Current studies are under way to sample more of the Eastern Pacific and other regions to encounter the missing taxa.

The Concerted Discovery Strategy (CDS) as initially described [5] and expanded upon by Olivera and Teichert [9] using α-conotoxins as a model, demonstrates the importance of understanding the phylogeny of the Conoidea when targeting novel bioactive compounds. Paramount to the strategy is the fact that the genes that encode venom toxins are rapidly evolving, to reflect changes in ecological niches. This ebb and flow between genes and the surrounding environment results in a diversification of toxins. One of the keys to understanding this diversifying selection process is to reliably reconstruct the phylogeny of the group and use it as a roadmap for the discovery of peptide toxins with therapeutic applications.

## 5. Conclusion

The Terebridae are a promising family within the Conoidea. Similar to cone snails terebrids possess venom peptide toxins that appear rich in variety and functional applications (Table 1). Preliminary results conducting biochemical [26] and molecular [27] characterization of teretoxins indicate they are very similar in structure to cone snail toxins. Teretoxins thus far identified appear to be larger than conotoxins (≥ 40 amino acids) and do not have posttranslation modifications, a feature commonly found in conotoxins. The lack of posttranslation modifications makes teretoxins an attractive target for analysis using mass spectrometry. Recently Ueberheide and colleagues [47] developed a mass spectrometry approach for elucidating toxin sequences from cone snails that utilizes the electron-transfer dissociation (ETD) method for tandem mass spectrometry. ETD is used to increase sequence coverage and improve mass detection to limits well beyond those of Edman sequencing and previous mass spectrometry methods. While limited by the current high cost of advanced mass spectrometry hardware, this technique appears to be a viable complement to the Concerted Discovery Strategy (CDS), and can be used both to confirm the expression and characterization of newly discovered teretoxins. While thus far applied only to cone snail toxins, the ETD inspired method also holds promise for identifying the primary amino-acid sequences of peptide toxins from terebrids and other venomous organisms. In addition, recombinant techniques such as the recently described tethered-toxin approach [48-51] facilitate the synthesis and folding of larger peptidic toxins.

Although not traditionally the molecular compound of choice for drug discovery, peptides, and especially peptidic toxins, are becoming increasingly important in the development of novel drug discovery pipelines. The N-type calcium (Ca^2+^) channel analgesic ziconotide, the first conotoxin drug, is striking for the molecular target and function combination it identified [19]. Prior to ziconotide's discovery Ca^2+ ^channels were not readily recognized as targets for pain alleviation. Similar to Ziconotide, an ω conotoxin, several other conotoxin families including, μ-conotoxins, which target voltage-gated Na^+ ^channels, k- and kM-conotoxins, which target K^+ ^channels, and conantokins, which target NMDA receptors, are under various stages of pharmaceutical development [5,20,52]. The potential applications of these conotoxins vary from pain, to epilepsy, and cardioprotective agents. In addition to conotoxins, peptidic toxins from scorpions, snakes and spiders, such as candoxin (Alzheimer's disease) [53], and α-Bgtx (myasthenic autoimmune response) [54,55] are making an impact in pharmacological developments. These peptides and the organisms that produce them are instrumental in identifying the next generation of therapeutics.

A discovery strategy such as CDS, which takes into account the divergent characteristic of peptide toxins from biodiverse organisms, paired together with current advances in peptide/proteomics, genomic and bioinformatic technologies provides a paradigm for investigating peptidic natural products that significantly enhances the identification of pharmacologically useful bioactive compounds. Current integrative initiatives that utilize ecological, genomic, proteomic, and functional activity based data of toxins, such as the cone snail genome project for health, CONCO http://www.conco.eu, and Venomics [56], will be useful in deciphering the potential and challenges ahead for terebrid toxin characterization.

## Authors' contributions

NP and MH read and approved the manuscript.
